# Changes of thyroid antibodies after thermal ablation of thyroid nodules: a retrospective study

**DOI:** 10.3389/fendo.2025.1689115

**Published:** 2025-10-21

**Authors:** Song Li, Ying Wei, Zhen-long Zhao, Li-li Peng, Yan Li, Ming-an Yu

**Affiliations:** Department of Interventional Ultrasound Medicine, China-Japan Friendship Hospital, Beijing, China

**Keywords:** thermal ablation, thyroid nodules, autoimmune thyroid disease, chronic lymphocytic thyroiditis, thyroid antibodiess

## Abstract

**Context:**

Thermal ablation (TA) is increasingly used as a minimally invasive treatment for thyroid nodules; however, its influence on thyroid autoimmunity remains unclear.

**Objective:**

To investigate longitudinal changes in thyroid autoantibodies and identify relevant risk factors.

**Methods:**

This retrospective study included 222 patients who underwent TA between April 2020 and September 2023. Serum levels of anti-thyroglobulin antibody (TGAb), anti-thyroid peroxidase antibody (TPOAb), and thyrotropin receptor antibody (TRAb) were measured at baseline and at 1, 6, 12, and 24 months post-ablation. Antibody trajectories, abnormality rates, and associated risk factors were analyzed.

**Results:**

TGAb and TRAb levels increased significantly at all post-ablation time points compared to baseline (all p < 0.05), while TPOAb showed a delayed but significant elevation beginning at 6 months. New-onset antibody positivity was observed in 16.2% of patients, including 9.0% with transient elevations, 4.1% with persistent positivity, and 3.2% with late-onset elevation. Among patients with transient elevations, 93.1% normalized within 24 months. At the 1-month follow-up, patients with benign nodules had more frequent antibody abnormalities than those with papillary thyroid carcinoma (9.5% *vs*. 2.7%, p = 0.045), although no significant differences were observed at subsequent time points. Multivariate analysis identified papillary thyroid carcinoma (OR = 7.70, p = 0.035), baseline TGAb (OR = 1.08, p < 0.001), and baseline TPOAb (OR = 1.12, p = 0.023) as independent predictors of post-ablation antibody abnormalities. ROC analysis demonstrated that a baseline TGAb level ≥17.32 IU/mL had moderate predictive value (AUC = 0.746), with a specificity of 85.5% and a negative predictive value of 91.9%.

**Conclusion:**

Thermal ablation was associated with transient increases in thyroid autoantibodies. A baseline TGAb level ≥17.32 IU/mL, elevated TPOAb, and a diagnosis of papillary thyroid carcinoma are associated with increased risk.

## Introduction

Thyroid nodules are a common clinical finding, identified in up to 65% of the general population using high-resolution ultrasonography (1). While most nodules are benign and asymptomatic, intervention is indicated when malignancy is suspected or when nodules cause compressive symptoms or cosmetic concerns (2, 3). In recent years, TA techniques—such as radiofrequency ablation (RFA) and microwave ablation (MWA)—have emerged as minimally invasive alternatives to surgery for the treatment of benign and select low-risk malignant thyroid nodules (4, 5). Compared to conventional thyroidectomy, TA offer several advantages, including outpatient feasibility, a lower complication rate, preservation of thyroid function, and faster recovery (6–8).

Although the efficacy of TA in achieving significant nodule volume reduction and local tumor control is well established, its impact on thyroid autoimmunity remains poorly understood. Thermal injury to thyroid tissue may expose or release thyroid-specific autoantigens, potentially triggering or exacerbating autoimmune responses (9). Autoantibodies such as anti-thyroid peroxidase antibody (TPOAb), anti-thyroglobulin antibody (TGAb), and thyrotropin receptor antibody (TRAb) are key serological markers of autoimmune thyroid diseases, including Hashimoto’s thyroiditis and Graves’ disease (10).

However, current evidence regarding the immunologic impact of TA remains limited and somewhat inconsistent. One study reported minimal changes in autoantibody levels at one month post-ablation (11), whereas another small-scale study observed a transient rise in TPOAb and TGAb levels that subsequently normalized (12). In contrast, a large study identified pre-existing Hashimoto’s thyroiditis as an independent risk factor for developing post-ablation thyroid dysfunction, underscoring the potential prognostic significance of baseline autoimmune status (13).

Given these uncertainties, further investigation is warranted to elucidate the long-term behavior of thyroid autoantibodies following TA and to identify relevant risk factors. This study aimed to comprehensively characterize longitudinal changes in TGAb, TPOAb, and TRAb levels after TA of thyroid nodules. We also examined the associations between clinical and procedural factors—such as nodule pathology, ablation technique, nodule size, and baseline antibody levels—and the development of post-ablation antibody abnormalities. In addition, the predictive value of baseline antibody levels was evaluated using receiver operating characteristic (ROC) analysis to support risk-stratified follow-up strategies. Our objective was to improve understanding of the immunological sequelae of TA and to inform evidence-based post-ablation surveillance.

## Materials and methods

This retrospective study was conducted in accordance with the Declaration of Helsinki and was approved by the Ethics Committee of the China-Japan Friendship Hospital (Approval No.: S2019-283-02). Prior to TA, all patients were fully informed of the potential risks, and written informed consent was obtained from each patient for both the procedure and the anonymous use of clinical and imaging data for publication. To protect patient privacy, the informed consent form is not publicly available.

### Patient selection

Inclusion criteria were as follows (1): cytological or pathological confirmation of benign thyroid nodules or papillary thyroid carcinoma (PTC) (2); patients who were either unsuitable for surgery or declined surgery and elected to undergo TA and (3) completion of uninterrupted follow-up visits at 1, 6, 12, and 24 months after ablation. Exclusion criteria included: (1) a history of thyroidectomy or prior TA; (2) a known diagnosis of autoimmune thyroid disease (e.g., Hashimoto’s thyroiditis or Graves’ disease); or (3) incomplete clinical or follow-up data.

### Pre-ablation assessment

All patients underwent ultrasound-guided fine-needle aspiration or core needle biopsy to confirm pathological diagnosis. Ultrasound imaging was used to document nodule characteristics, including size, number, location, margins, composition, calcification, and vascularity. Laboratory assessments included complete blood count, coagulation profile, and thyroid function tests. Baseline serum levels of TPOAb, TGAb, and TRAb were also measured. All antibody measurements were performed using the same standardized electrochemiluminescence immunoassay platform (Roche, Germany), with routine internal quality control procedures. Inter-assay variability and lot-to-lot drift were not systematically assessed, which is acknowledged as a limitation.

### Thermal ablation procedure

All patients underwent preoperative contrast-enhanced ultrasound (CEUS) to evaluate tumor vascularity and margins. TA was performed under local anesthesia using either a 17G internally cooled antenna (Nanjing ECO Intelligent Basic Microwave Tumor Ablation System) or a Cool-tip Radiofrequency Ablation System (Covidien), both equipped with a 7 mm active tip. In accordance with our prior reports, hydrodissection was utilized to safeguard adjacent critical structures such as the recurrent laryngeal nerve, trachea, and esophagus (14).

For malignant nodules, multi-point ablation and margin-expansion (2mm) techniques were used to ensure comprehensive treatment (15). For benign nodules, the entire lesion was required to be encompassed within the non-enhancing ablation zone. Median ablation time was 112s (IQR 69–197) with a power of 30 W. The completeness of ablation was confirmed by immediate post-procedure contrast-enhanced ultrasound. No corticosteroids or NSAIDs were routinely administered peri-procedurally. CEUS was repeated immediately post-procedure to confirm adequate coverage (16).

### Post-ablation assessment and follow-up

Follow-up assessments were conducted every six months during the first year and annually thereafter. Each follow-up included thyroid function tests (FT3, FT4, TSH), autoantibody measurements (TGAb, TPOAb, TRAb), and neck ultrasonography. For patients with malignant nodules, annual neck or chest CT scans were also performed to detect potential lymph node or distant metastases. When metastatic disease was suspected, fine-needle aspiration or core needle biopsy was performed for histological confirmation (17).

### Serological assessment and outcome classification

Serum levels of TGAb, TPOAb, and TRAb were measured at baseline and at each follow-up visit in the hospital’s endocrine laboratory using standardized electrochemiluminescence immunoassays. The reference ranges were TGAb <115 IU/mL, TPOAb <34 IU/mL, and TRAb <1.75 IU/L (13); values exceeding these thresholds were considered positive. To ensure consistency, all samples were analyzed on the same assay platform throughout the study period.

Post-ablation antibody responses were categorized into four groups based on the longitudinal patterns of TGAb, TPOAb, and TRAb: (1) Persistent elevation – antibody positivity observed at multiple time points that did not normalize by the end of follow-up. (2) Transient elevation – antibody levels became positive after ablation but returned to normal within 24-month after ablation. (3) Late-onset elevation – all antibody levels remained within the normal range through 12 months, with antibodies becoming positive for the first time at 24 months. (4) Stable seronegativity – all antibody levels remained within the normal range at all follow-up time points.

Patients who met the criteria for persistent, transient, or late-onset elevation of any antibody were classified as having an antibody abnormality, whereas those who remained seronegative for all antibodies throughout follow-up were considered antibody-normal. By study design, all patients were seronegative at baseline; thus, any post-ablation antibody elevation was considered a new-onset event, rather than a continuation of pre-existing autoimmunity.

### Statistical methods

Statistical analyses were performed using R (version 4.2.2), SPSS (version 26.0), and Stata (version 17.0). Continuous variables were expressed as mean ± standard deviation (SD) or median with interquartile range (IQR), as appropriate. Between-group comparisons of continuous variables were conducted using the Mann–Whitney U test. Categorical variables were compared using Pearson’s chi-square test or Fisher’s exact test, depending on expected cell counts.

Paired comparisons were conducted using the paired t-test or Wilcoxon signed-rank test to evaluate changes in antibody levels at each follow-up time point relative to baseline. A multivariable logistic regression model was constructed to identify independent predictors of post-ablation antibody abnormalities. Receiver operating characteristic (ROC) curve analysis was used to evaluate the predictive performance of baseline antibody levels. No adjustment for multiplicity was performed given the exploratory nature of this analysis. A two-sided p-value <0.05 was considered statistically significant.

## Results

### Baseline characteristics

Baseline characteristics of the 222 patients are summarized in **Table 1**. The cohort comprised 156 females (70.3%) and 66 males (29.7%), with a median age of 42 years (IQR 34–53). Papillary thyroid carcinoma was the diagnosis in 148 patients (66.7%), while 74 patients (33.3%) had benign thyroid nodules.

**Table 1:** Patient demographics and baseline characteristics.

Characteristic	N = 222
Sex	
Female	156 (70.27%)
Male	66 (29.73%)
Age (years)	42 (34, 53)
Nodules type	
Benign thyroid nodules	74 (33.33%)
Papillary thyroid carcinoma	148 (66.67%)
BRAF V600E mutation	
Negative	94 (42.34%)
Positive	128 (57.66%)
Maximum diameter of the nodule (cm)	0.80 (0.50, 1.90)
Location of the nodules	
Isthmus	9 (4.05%)
Left lobe	98 (44.14%)
Right lobe	115 (51.80%)
CEUS	
Hyper-enhancement	106 (47.75%)
Hypo-enhancement	89 (40.09%)
Iso-enhancement	27 (12.16%)
Calcification	
None	181 (81.53%)
Yes	41 (18.47%)
Composition	
Mixed cystic-solid	24 (10.81%)
Solid	198 (89.19%)
Margin	
Irregular	136 (61.26%)
Smooth	86 (38.74%)
Number of nodules treated with TA	
Multiple	94 (42.34%)
Single	128 (57.66%)
Technique of TA	
Microwave ablation	193 (86.94%)
Radiofrequency ablation	29 (13.06%)
Ablation time (s)	112 (69, 197)
Ablation power (W)	30 (30, 30)

TA, thermal ablation; CEUS, contrast-enhanced ultrasound.

### Changes of thyroid antibodies after thermal ablation

**Figure 1:**
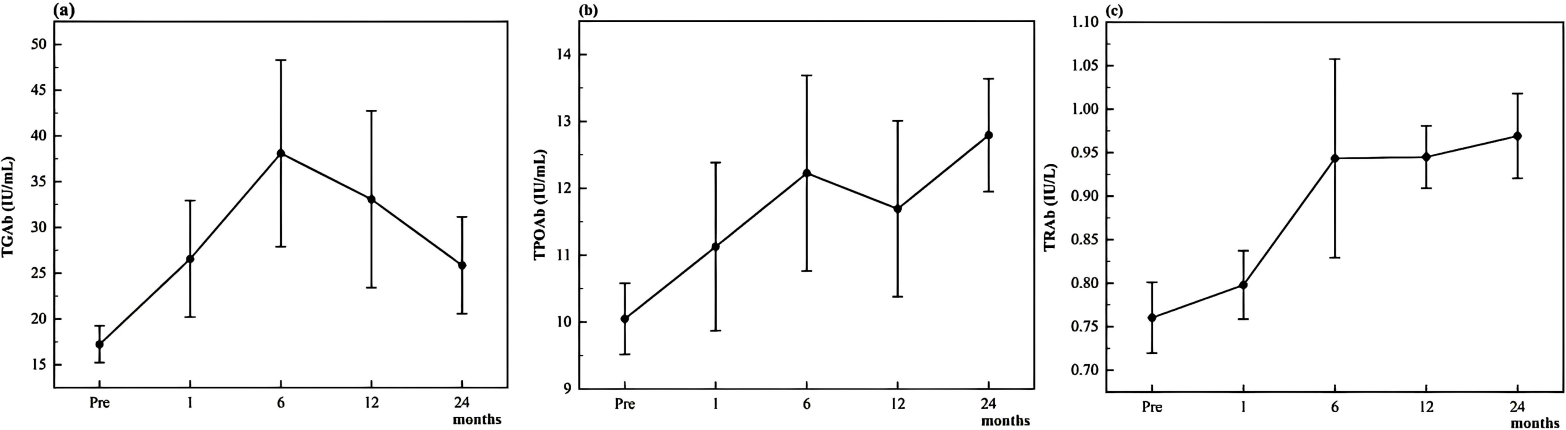
Changes of thyroid antibodies TGAb **(a)**, TPOAb **(b)**, and TRAb **(c)** during follow-up.

**Figure 1** illustrates the longitudinal trends in mean antibody levels over time. At baseline, the mean levels of TGAb, TPOAb, and TRAb were 17.25 ± 15.18 IU/mL, 10.05 ± 4.02 IU/mL, and 0.76 ± 0.31 IU/L, respectively. TGAb and TRAb levels were significantly elevated at all post-ablation time points (1, 6, 12, and 24 months) compared to baseline (all p < 0.05). In contrast, TPOAb levels did not show a significant increase until 6 months post-ablation, after which they remained significantly higher than baseline at both 12 and 24 months (all p < 0.05).

### Serological response patterns

Of the 222 patients, 36 (16.2%) developed new-onset thyroid autoantibody positivity during the 24-month follow-up period. The distribution of antibody abnormalities by type and time point is summarized in **Table 2**. Among these, 9 patients (4.1%) exhibited persistent antibody elevation, 20 patients (9.0%) showed transient post-ablation antibody positivity, and 7 patients (3.2%) demonstrated late-onset antibody elevation, first detected at the 24-month visit. The remaining 186 patients (83.8%) maintained stable seronegativity throughout follow-up.

**Table 2:** Abnormalities of different antibody types at each follow-up time point.

Follow-up time	TGAb antibody abnormalities	TPOAb antibody abnormalities	TRAb antibody abnormalities	Total
1-month	9 (4.05%)	3 (1.35%)	0 (0%)	12
6-months	17 (7.66%)	5 (2.25%)	3 (1.35%)	25
12-months	12 (5.41%)	3 (1.35%)	4 (1.80%)	19
24months	9 (4.05%)	6 (2.70%)	3 (1.35%)	18
Total	47	17	10	74

**Figure 2:**
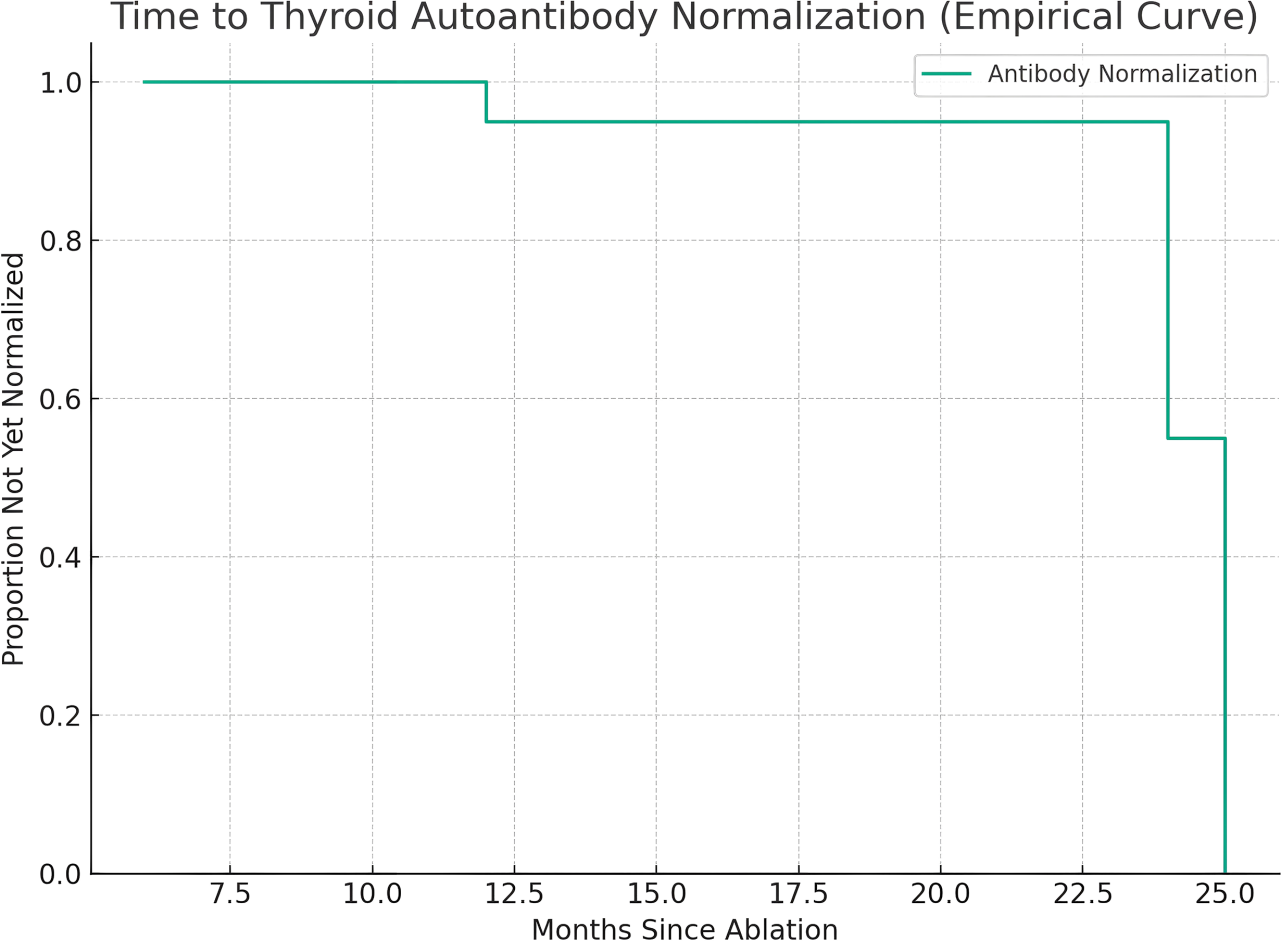
Kaplan–Meier analysis of time to recovery from transient antibody abnormalities.

Among those with transient elevations, the median time to antibody normalization was 12 months (IQR 6–24 months). **Figure 2** shows the Kaplan–Meier analysis of antibody normalization, which revealed that 93.1% of affected patients had returned to normal levels by 24 months. There was no significant difference in normalization rates between TGAb and TPOAb subtypes (log-rank p = 0.42).

### Subgroup analysis: benign thyroid nodules *vs*. papillary thyroid carcinoma

At the one-month follow-up, the rate of antibody abnormalities was significantly higher in patients with benign nodules compared to those with papillary thyroid carcinoma (9.5% *vs*. 2.7%, p = 0.045). However, no significant differences were observed between the two groups at subsequent follow-up time points (all p > 0.05). Details are presented in **Table 3**.

**Table 3:** Changes of antibodies after thermal ablation of benign thyroid nodules and papillary thyroid carcinoma.

Follow-up time	Benign thyroid nodules n = 74	Papillary thyroid carcinoma n = 148	P-value
1-month			0.045
normal	67 (90.5%)	144 (97.3%)	
abnormal	7 (9.5%)	4 (2.7%)	
6-months			0.533
normal	65 (87.8%)	134 (90.5%)	
abnormal	9 (12.2%)	14 (9.5%)	
12-months			0.734
normal	67 (90.5%)	136 (91.9%)	
abnormal	7 (9.5%)	12 (8.1%)	
24-months			0.142
normal	66 (89.2%)	140 (94.6%)	
abnormal	8 (10.8%)	8 (5.4%)	
Total			0.699
normal	61 (82.4%)	125 (84.5%)	
abnormal	13 (17.6%)	23 (15.5%)	

BTN, Benign thyroid nodules; PTC, Papillary thyroid carcinoma.

### Risk factors analysis of thyroid function abnormality

**Figure 3:**
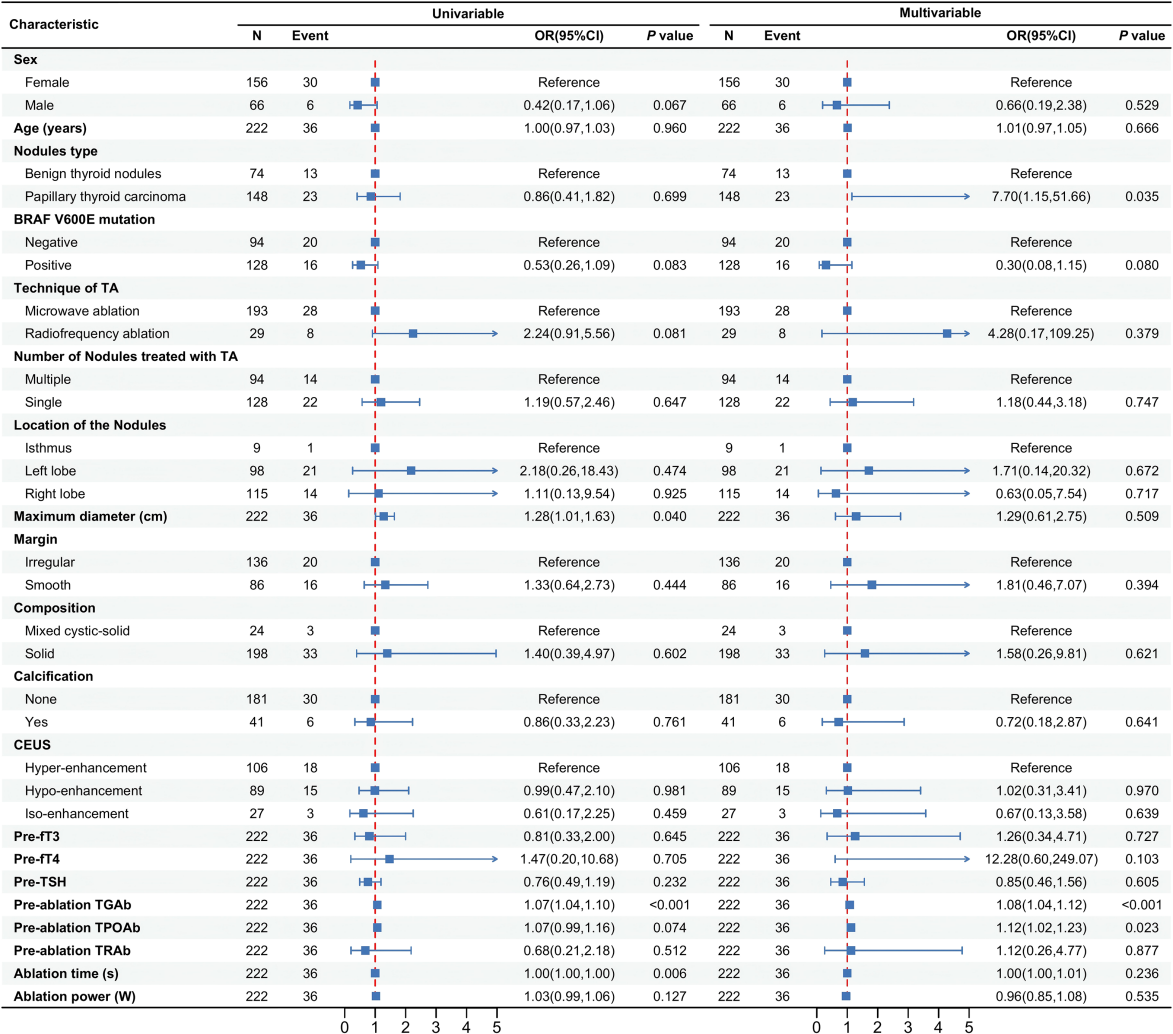
Analysis of risk factors for abnormal thyroid antibodies after ablation.

**Figure 3** presents the analysis of risk factors for abnormal thyroid antibody development following ablation. Multivariate logistic regression revealed that patients with papillary thyroid carcinoma (PTC) had a significantly higher risk of developing antibody positivity compared to those with benign thyroid nodules (BTNs) (OR = 7.70, 95% CI: 1.15–51.66, p = 0.035). Higher baseline levels of TGAb (OR = 1.08, 95% CI: 1.04–1.12, p < 0.001) and TPOAb (OR = 1.12, 95% CI: 1.02–1.23, p = 0.023) were also independently associated with increased risk of post-ablation antibody abnormalities.

Other variables—including gender, age, BRAF V600E mutation status, ablation technique, number of nodules treated, nodule location, maximum diameter, margin characteristics, composition, calcification, contrast-enhanced ultrasound (CEUS) pattern, thyroid function markers (fT3, fT4, TSH), TRAb levels, and ablation parameters (time, power)—were not significantly associated with antibody status (all p > 0.05).

### Predictive value of baseline antibody levels

**Figure 4:**
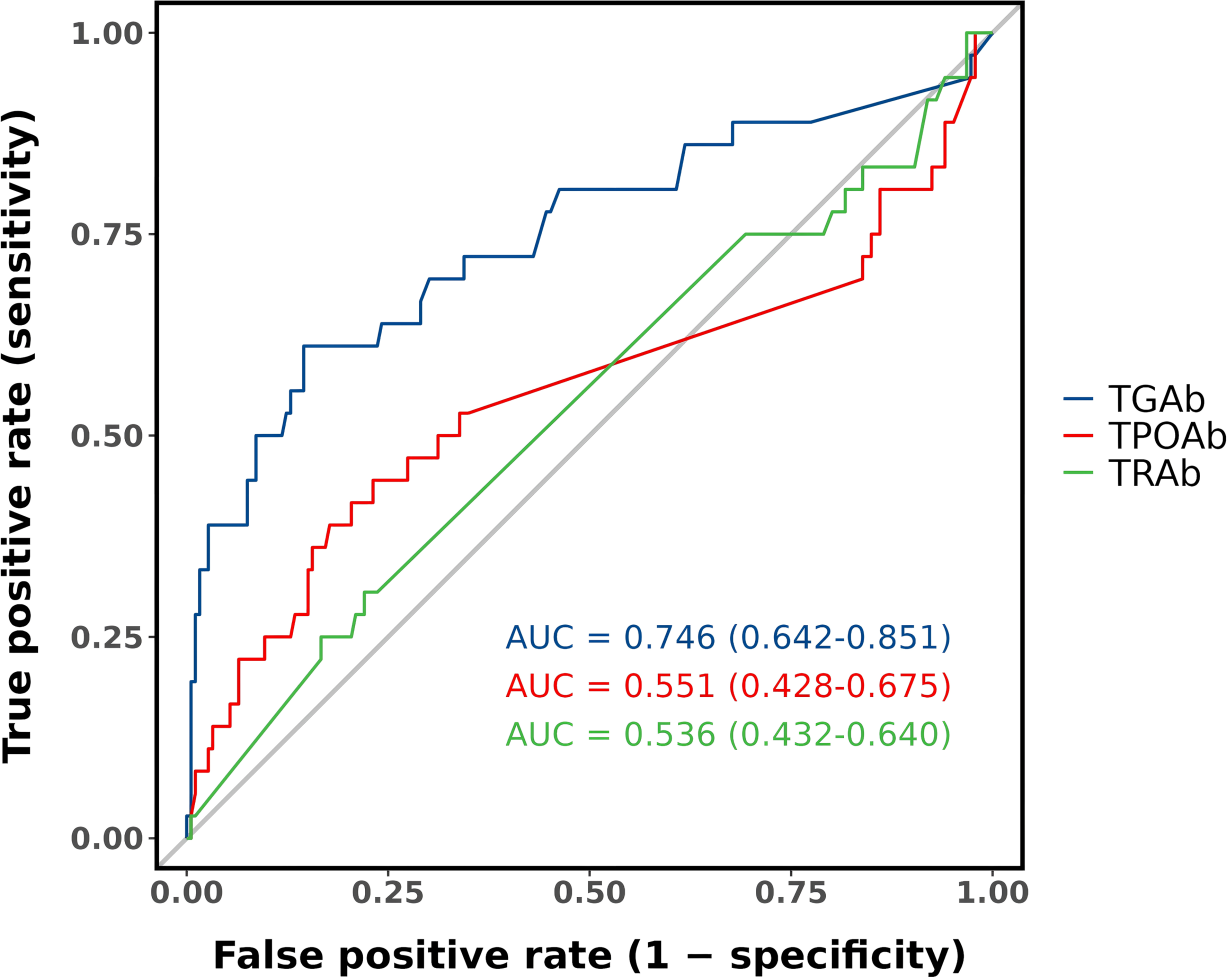
Predictive value of baseline antibody levels.

The predictive performance of baseline TGAb, TPOAb, and TRAb levels for post-ablation antibody abnormalities is shown in **Figure 4**. Receiver operating characteristic (ROC) analysis demonstrated that baseline TGAb had moderate predictive value for identifying patients at risk of developing antibody abnormalities after ablation (AUC = 0.746, 95% CI: 0.642–0.851). The optimal cut-off value for TGAb was 17.32 IU/mL, yielding a sensitivity of 61.1%, specificity of 85.5%, positive predictive value of 44.9%, negative predictive value of 91.9%, and an overall accuracy of 81.5%. In contrast, baseline TPOAb and TRAb levels exhibited poor predictive performance, with AUCs of 0.551 and 0.536, respectively—both approximating random classification.

## Discussion

This study provides new evidence that TA, though considered a localized intervention, may transiently influence thyroid autoimmunity. In this cohort of seronegative patients, 16.2% developed new-onset autoantibody positivity following TA, typically in the form of TGAb or TPOAb elevation. To our knowledge, this is one of the first studies to systematically track longitudinal antibody patterns up to 24 months post-TA and to quantify baseline predictors of immune response, including a TGAb threshold with clinical utility.

TGAb and TRAb levels increased significantly at all post-ablation time points, while TPOAb exhibited a delayed but persistent elevation beginning at 6 months. These temporal differences may reflect variations in antigen release kinetics, immune activation thresholds, or clearance rates among antibody types. The relatively low incidence of TRAb positivity (4.5%) is consistent with the absence of patients with Graves’ disease in this cohort.

Although post-ablation antibody abnormalities were not uncommon, they were generally transient: 93.1% of affected patients returned to seronegative status within 24 months, and no cases of overt autoimmune thyroid disease or thyroid dysfunction were observed. These findings suggest that TA-related autoantibody elevations are largely self-limiting and subclinical in the short to midterm.

Multivariate analysis identified three independent predictors of post-ablation antibody abnormalities: elevated baseline TGAb, elevated baseline TPOAb, and a diagnosis of papillary thyroid carcinoma (PTC). Importantly, these factors may reflect distinct immunologic mechanisms. The association between baseline TGAb/TPOAb and seroconversion suggests that even subclinical autoimmune activity may predispose patients to enhanced immunologic responses upon tissue injury. By contrast, the increased risk observed in patients with PTC may be related to tumor-specific factors, including increased antigen burden, peritumoral lymphocytic infiltration, or altered immune tolerance, as described in prior studies (18, 19).

Among the three markers assessed, TGAb demonstrated the strongest predictive performance, with a threshold of ≥17.32 IU/mL yielding high specificity (85.5%) and a negative predictive value of 91.9%. This suggests potential value in pre-ablation TGAb screening to identify patients at greater risk for serologic changes after ablation. TPOAb and TRAb, by contrast, showed weaker predictive utility in this context.

Interestingly, antibody abnormalities were more common at 1 month post-ablation in patients with benign thyroid nodules than in those with PTC. However, this early difference diminished over time, and the overall risk remained higher in the PTC group. Given the lack of mechanistic clarity, the early elevation in benign cases may reflect acute inflammatory responses to ablation or procedural variables (e.g., ablation volume), rather than sustained immunologic susceptibility. Further studies are needed to explore these differences.

The inclusion of both benign nodules and papillary thyroid carcinoma introduces biological heterogeneity. Although subgroup analyses were performed, the results should be regarded as exploratory given the relatively small sample sizes and the lack of adjustment for multiple comparisons. In addition, although more than half of PTC cases were BRAF V600E positive, we did not perform a stratified analysis by mutation status due to limited antibody-positive cases. Future studies should explore the potential relationship between BRAF mutation and antibody response.

Another potential factor influencing antibody changes is fine-needle aspiration (FNA), which was performed in all patients prior to ablation. Interestingly, we observed that a very small proportion of patients developed antibody abnormalities after FNA but before ablation, despite being seronegative at baseline, suggesting that FNA itself may occasionally induce minor antibody fluctuations. To minimize this potential confounding effect, our exclusion criteria eliminated patients with pre-existing autoimmune thyroid disease as well as those who presented with antibody abnormalities prior to ablation, including those likely related to FNA. Therefore, all patients included in this study were antibody-negative before ablation. Although we did not establish a dedicated FNA-only surveillance group, the clear temporal relationship of antibody elevation following ablation (TGAb and TRAb from 1 month, and TPOAb from 6 months) and the transient nature of most abnormalities strongly suggest that ablation-induced tissue injury is the predominant cause of these changes.

These findings may have practical relevance. While TA remains a safe and effective modality, clinicians should be aware that mild and transient autoantibody elevations can occur, especially in patients with elevated baseline TGAb or underlying PTC. Routine autoantibody screening may not be necessary for all patients; however, targeted pre-ablation testing—particularly for TGAb—may help guide follow-up strategies in higher-risk subgroups. These results support a risk-adapted rather than universal approach to post-ablation immune monitoring.

This study has several limitations. Its retrospective, single-center design may limit generalizability. Patients with known autoimmune thyroid disease and those with preoperative antibody abnormalities (potentially attributable to FNA) were excluded, which may have led to an underestimation of the overall immune response to TA. Antibody measurements were conducted at fixed intervals; interim fluctuations could not be assessed. No formal power calculation was performed prior to the study; however, our cohort of 222 patients is relatively large compared with previously published series. Nonetheless, the relatively small number of antibody-positive events may limit statistical power and stability of multivariate analyses. Although all patients were seronegative at baseline, we acknowledge that borderline subclinical antibody activity near the cutoff could not be completely excluded and may have influenced the results. Furthermore, the ROC analysis was not cross-validated, and calibration statistics were not assessed, thus the predictive performance should be interpreted with caution. Finally, although no overt thyroid dysfunction was observed, the long-term clinical significance of persistent TGAb or TPOAb elevations remains uncertain and warrants evaluation in larger, prospective studies with extended follow-up. The wide confidence intervals observed in logistic regression analyses reflect the small number of events and suggest model instability.

TA of thyroid nodules is associated with modest but detectable increases in thyroid autoantibody levels in a subset of patients, particularly those with PTC or subclinical pre-existing autoimmunity. These changes are usually transient and clinically silent. The proposed cutoff value for TGAb (≥17.32 IU/mL) may be a potential predictor of post-ablation antibody abnormalities, but this finding requires external validation before clinical application. These findings support the integration of individualized, risk-based antibody monitoring into the long-term management of patients undergoing TA.

## Data Availability

The datasets presented in this article are not readily available because The datasets generated and analyzed during this study are not publicly available due to patient privacy restrictions but can be accessed from the corresponding author upon reasonable request. All data provided will be anonymized to comply with ethical standards. Requests to access the datasets should be directed to M-AY: yma301@163.com.
